# Marine archaea and archaeal viruses under global change

**DOI:** 10.12688/f1000research.11404.1

**Published:** 2017-07-27

**Authors:** Roberto Danovaro, Eugenio Rastelli, Cinzia Corinaldesi, Michael Tangherlini, Antonio Dell'Anno

**Affiliations:** 1 Stazione Zoologica Anton Dohrn, Villa Comunale, Naples, Italy; 2 Department of Life and Environmental Sciences, Polytechnic University of Marche, Ancona, Italy; 3 Department of Sciences and Engineering of Materials, Environment and Urbanistics, Polytechnic University of Marche, Ancona, Italy

**Keywords:** Marine archaea, archaeal viruses, Thaumarchaeota

## Abstract

Global change is altering oceanic temperature, salinity, pH, and oxygen concentration, directly and indirectly influencing marine microbial food web structure and function. As microbes represent >90% of the ocean’s biomass and are major drivers of biogeochemical cycles, understanding their responses to such changes is fundamental for predicting the consequences of global change on ecosystem functioning. Recent findings indicate that marine archaea and archaeal viruses are active and relevant components of marine microbial assemblages, far more abundant and diverse than was previously thought. Further research is urgently needed to better understand the impacts of global change on virus–archaea dynamics and how archaea and their viruses can interactively influence the ocean’s feedbacks on global change.

## Recent insights on marine archaea and their respective viruses

**Figure 1.**
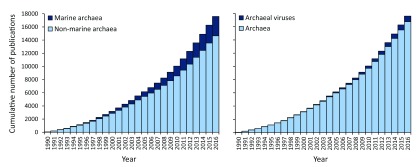
Number of publications regarding archaea, subdivided into publications on marine or non-marine archaea (left chart) and publications on archaea or on viruses of archaea (right chart), as searched through the Web of Science database. The following keywords were used in the searches for archaea: *archaea or archaebacteria or archaeobacteria*. The following keywords were used in the searches for marine archaea: *marine archaea or ocean archaea or sea archaea,* also using the terms *archaebacteria or archaeobacteria*. The following keywords were used in the searches for viruses of archaea: *archaea virus or archaebacteria virus or archaeobacteria virus*. Research on archaea has increased over the past few decades, but relatively little focus has been directed towards marine archaea and archaeal viruses, despite the current compelling evidence of their relevant role at the global level.

Research on archaea has increased exponentially over the last few years, but marine archaea and the viruses able to infect them have received little attention despite their global relevance ^1– 6^ ( Figure 1). Moreover, most of the current knowledge on archaea and archaeal viruses is based on culturable extremophiles inhabiting peculiar high-temperature, high-salinity, or low-pH environments ^7– 9^, while the archaeal taxa most represented in the ocean remain almost completely uncultured ^10– 16^, as well as their viruses ^17^. Several new archaeal phyla have been described in the last 15 years thanks to gene surveys, metagenomics studies, and single-cell next-generation sequencing projects ^16, 18– 22^. While the earliest archaeal phylogenetic trees reported only two phyla (i.e. the Crenarchaeota and Euryarchaeota), the current view of the taxonomic and functional diversity of archaea has greatly expanded. Besides Euryarchaeota, three additional archaeal clades have been recently proposed: the TACK superphylum (including Crenarchaeota, Korarchaeota, Thaumarchaeota, Aigarchaeota, and Bathyarchaeota), the DPANN superphylum (including archaeal Richmond Mine acidophilic nano-organism [ARMAN], Diapherotrites, Nanohaloarchaea, Parvarchaeota, Aenigmarchaeota, and Nanoarchaeota), and the ASGARD superphylum (including Lokiarchaeota, Thorarchaeota, Odinarchaeota, and Heimdallarchaeota) ^16, 23– 27^. Moreover, findings based on current culture-independent molecular approaches point out that a large fraction of archaeal diversity is still awaiting discovery ^28^.

Marine ecosystems host 3.9×10 ^30^ prokaryotic (i.e. bacterial and archaeal) cells and 4.3×10 ^31^ viruses ^29^. These components represent ~90% of the global microbial abundance and largely contribute to organic matter cycling and biogeochemical processes on a global scale ^29, 30^. Most of such microbes live in deep-sea ecosystems (i.e. waters and sediments below 200 m water depth), which cover more than 65% of the Earth’s surface and represent 95% of the biosphere volume ^31, 32^. Archaea are ubiquitous and abundant in marine ecosystems. Although bacteria tend to outnumber archaea in the world’s oceans, archaea make an important contribution to microbial biomass in deep waters (with abundances equivalent to those of bacteria at depths of >1000 m) and in surface and subsurface marine sediments ^3, 31^. Different archaeal taxa can be numerically dominant in specific environmental conditions. In oxygenated waters and sediments, four major groups of archaea prevail, including marine group (MG)-I Thaumarchaeota and MG-II, MG-III, and MG-IV Euryarchaeota ^15^, while novel archaeal taxa have been recently identified in anoxic ecosystems including marine subsurface sediments ^16, 27^. MG-I Thaumarchaeota are among the most abundant microbes in the deep ocean, and they play key roles in global C and N cycles through CO _2_ fixation coupled with ammonium/ammonia oxidation, which can generate the greenhouse gas nitrous oxide (N _2_O) as a by-product ^3, 11, 14, 33^. MG-II Euryarchaeota generally prevail in surface waters ^15, 34^ and can display heterotrophic/photoheterotrophic lifestyles ^35, 36^, while the physiology and metabolism of MG-III and MG-IV Euryarchaeota, preferentially inhabiting the ocean interior at relatively low abundances, still remain poorly understood ^15, 37^. In subsurface and anoxic sediments, still poorly resolved archaeal groups, such as anaerobic methanotrophic archaea (ANME) and members of the deep sea archaeal group (DSAG) and of the miscellaneous Crenarchaeota group (MCG), can account for a large fraction of prokaryotic standing stocks ^25, 38– 40^ and are thought to significantly contribute to biogeochemical cycles and global ecosystem functioning ^21, 26, 27, 41^.

Concerning archaeal viruses, a putative provirus has been identified in a recently isolated MG-I thaumarchaeon ^42^, and DNA sequences of putative viruses infecting MG-I Thaumarchaeota are abundant in seawater and sediments ^3, 4, 43– 45^. Increasing evidence suggests that marine viruses infecting members of other dominant archaeal taxa are widespread and likely highly abundant both in the water column (e.g. putative viruses of pelagic MG-II Euryarchaeota) ^5, 6^ and in sediments (e.g. putative viruses of anaerobic methane-oxidizing euryarchaea ^46^ and other still-unclassified archaeal viruses ^47^). All of these viruses are still uncultured ^17^, and the virus–archaea interactions occurring in marine ecosystems remain largely unknown ^3, 4, 48^.

## Impacts of climate changes on marine archaea

Since the beginning of the industrial revolution, anthropogenic activities have progressively enhanced terrestrial fluxes of greenhouse gases, increasing atmospheric concentrations of CO _2_, CH _4_, and N _2_O by 40%, 150%, and 20%, respectively ^49, 50^. This process has triggered climate changes causing warming, oxygen depletion, and acidification of the oceans as well as altered precipitation regimes, increased ice melting, and shifting patterns (generally, a decrease) of global primary production and carbon export to the ocean interior. All these changes have been reported to influence the biodiversity and functioning of marine ecosystems ^51– 55^.

Marine archaea are key actors in the cycling of all the aforementioned greenhouse gases ^26, 33, 56– 58^. Our knowledge on the potential consequences of global climate changes on archaea is very limited and almost entirely based on studies of MG-I Thaumarchaeota ^11, 13, 33^. Indeed, the recent success in culturing several MG-I Thaumarchaeota as pure isolates or in enriched mixed cultures has provided the first insights into their responses to changes in seawater temperature, oxygen concentrations, and pH ^11, 13, 14, 33, 56^. The emerging view suggests high functional diversity and metabolic plasticity within the MG-I Thaumarchaeota, including members with different sensitivities to seawater warming, acidification, and oxygen depletion ^11, 14, 33^. Thus, specific MG-I Thaumarchaeota more adapted to such conditions could be competitively advantaged in future scenarios of global change ^11, 33^.

Global warming is expected to have a stronger impact on marine ecosystems at high latitudes ^51, 59^, where pelagic and benthic MG-I Thaumarchaeota are particularly abundant and highly metabolically active ^3, 45, 60, 61^. While primary production is expected to decrease at tropical and mid-latitudes, an increase is expected at high latitudes ^51, 62– 64^. If confirmed, these shifts will alter the quantity and quality of food supply to the seafloor ^65^, with downstream consequences on organic matter remineralization and supply of ammonia needed for sustaining the metabolism of nitrifying MG-I Thaumarchaeota. On one hand, such changes could have a differential impact on MG-I Thaumarchaeota at different latitudes ^45^. On the other hand, inter-strain hallmarks of different members within MG-I Thaumarchaeota, including chemotaxis, motility, and versatility in organic substrate utilization, might be factors able to influence their relative distribution under future scenarios of global change ^14^. Current evidence suggests that the shifts in food supply caused by global change ^51, 65^ could influence also the distribution and abundance of MG-II Euryarchaeota, which are believed to be heterotrophs ^15, 35, 36^ and whose abundance in benthic deep-sea ecosystems can be controlled by the availability of organic matter ^45^. Nonetheless, the lack of available culturable strains for this and other newly discovered archaeal taxa ^15, 16^ limits our ability to predict their metabolic/physiological response to global changes.

Recent studies have provided the first insights into the possible effects of temperature changes on archaeal assemblages. While manipulative experiments have suggested no significant effects of temperature shifts from 8 to 20°C on the rates of ammonia oxidation by archaea ^66^, temperature itself has been shown to be a significant macroecological driver of the global patterns of distribution of MG-I Thaumarchaeota in benthic deep-sea ecosystems ^45^. In polar ecosystems, the enhanced ice melting due to global warming has the potential to influence the composition and relative abundance of marine archaea by releasing ice-associated microbes ^67, 68^ and favoring specific MG-I Thaumarchaeota pre-adapted to grow at lower salinity ^11^. Moreover, as nitrification in Thaumarchaeota is dependent upon oxygen levels and is inhibited by anoxic conditions ^33^, the expansion of oxygen-depleted marine zones induced by warming and eutrophication can contribute to influence on the composition and functioning of archaeal assemblages ^69– 71^. However, the impacts of such changes, including the relative feedbacks of different archaeal taxa and the consequences on carbon and nutrient cycling, remain virtually unexplored ^71^.

## Virus–archaea interactions in the oceans under global change

Daily virus-induced mortalities of marine prokaryotes are in the range of 0.1–40% of the standing stock ^3, 72, 73^, implying that every year approximately 10 ^30^ to 10 ^32^ prokaryotic cells are infected and killed by viruses in marine ecosystems ^29^. However, the extent to which viruses specifically impact archaea in the oceans is largely unknown, and this represents a significant gap for a better comprehension of the functioning of the world’s oceans.

The relative contribution of archaea to the prokaryotic stock has been reported to increase with increasing water column depth and along the vertical profile of the sediment ^15, 39^. Also, viruses are suggested to play more relevant roles delving deeper in the ocean interior and in the subsurface, where the virus-to-prokaryote abundance ratios and the representation of virus-related DNA sequences in metagenomes is typically higher ^74– 79^. Recent studies provide evidence of a high virus-induced mortality on archaea (mainly on MG-I Thaumarchaeota) in benthic deep-sea ecosystems, resulting in the release of ~0.3 to 0.5 gigatons of carbon per year globally ^3^. MG-I Thaumarchaeota use CO _2_ to produce biomass, while viruses kill them, releasing their labile cellular content, thus enhancing organic matter remineralization and respiration processes of uninfected heterotrophic microbial components ^3^. In turn, the stimulation of heterotrophic processes can enhance nitrogen regeneration processes, supplying 30 to 60% of the ammonia required to sustain archaeal chemoautotrophic C production in deep-sea sediments ^3^. Understanding the factors able to influence this complex network of microbial CO _2_-consuming and CO _2_-producing processes will provide insights into the ability of the oceans to act as source or sink for this important greenhouse gas.

Since viral replication is linked with host metabolic state, impacts of global changes on the physiology and metabolism of archaea will likely influence also virus–archaea dynamics ^80^. At the same time, the success of different archaeal taxa in the future, based on their ability to adapt to the predicted scenarios of global change, will likely influence the relative importance of their viruses. For instance, the changes in the activity, diversity, and distribution of marine bacteria and archaea due to the spreading of oxygen-depleted pelagic and benthic zones ^69, 70^ will likely determine also shifts in the assemblages of viruses, whose role can be particularly relevant in low-oxygen conditions ^71, 81^. Similarly, the opposite effects of global changes on primary production and carbon export at high versus middle/tropical latitudes will likely influence in a different way the activity, diversity, and distribution of archaeal viruses on the basis of the different sensitivity to temperature and food availability of their respective archaeal hosts.

Evidence at different latitudes suggests that viral responses to increasing temperature (e.g. viral production rates) display increasing or decreasing trends in polar and in temperate systems, respectively ^80^. Further research is needed to understand the role of viruses of archaea in explaining such trends, especially at high latitudes, which host some of the most rapidly warming oceanic regions, as well as large numbers of archaea ^3, 45, 60, 61^.

Recent studies carried out in marine ecosystems have pointed out the importance of lysogenic viral infections, in which a provirus coexists in its host until replication is induced, leading or not to cell lysis mainly depending on environmental factors ^82^. Taking into account lysogenic viral infections can be critical to predicting changes in bacterial dynamics under scenarios of global change, while such information is currently lacking for archaea ^83^. Indeed, despite putative proviruses having been identified in Thaumarchaeota ^42^, the relative importance of lytic versus lysogenic virus–archaea dynamics and their response to the environmental changes brought on by global change are still largely unknown.

Considering that viruses can confer novel functions to their host through lateral gene transfer and/or expression of virus-encoded auxiliary metabolic genes ^84, 85^, we should take into consideration the possibility that such virus–host interactions could influence hosts’ responses to global change. Recently, viral auxiliary metabolic genes have been proposed to modulate ammonia oxidation in Thaumarchaeota ^4^, suggesting that future studies on virus–archaea interactions will contribute to clarifying ecological and biogeochemical processes of global relevance.

Overall, global change will alter a wide variety of marine microbial-mediated processes, including virus–archaea interactions, which remain as-yet largely unknown despite their significant ecological and biogeochemical implications. Deepening the knowledge on marine virus–archaea dynamics can thus significantly contribute to understanding the ocean’s feedbacks on global climate change. The biology, ecology, and evolution of archaea and archaeal viruses is one of the most intriguing and timely topics in microbial ecology, and research perspectives on the marine realm can be extended to freshwater and terrestrial ecosystems, in which archaea are also widespread and diversified ^86– 90^.
